# Role of Family Medicine in Integrative Prevention and Management of Oncologic Diseases in Romania

**DOI:** 10.3390/healthcare14162556

**Published:** 2026-08-15

**Authors:** Polliana Mihaela Leru, Vlad Florin Anton, Cristina Cercel, Irina Anca Eremia, Sergiu Marian Cazacu, Carmen Daniela Neagoe

**Affiliations:** 1 Clinical Department 5, Carol Davila University of Medicine and Pharmacy, 202021 Bucharest, Romania; polliana.leru@umfcd.ro (P.M.L.);; 2 Colentina Clinical Hospital, 020125 Bucharest, Romania; 3 The University Emergency Hospital of Bucharest, 050098 Bucharest, Romania; 4 Department of Gastroenterology, University of Medicine and Pharmacy of Craiova, 200349 Craiova, Romania

**Keywords:** family medicine, healthcare policy, oncologic patients, primary healthcare, multidisciplinary team

## Abstract

The incidence of neoplastic disorders worldwide has steadily increased, and Romania is no exception, showing a substantial rise in active oncologic surveillance cases. Family physicians are essential to the multidisciplinary team, providing screening, early diagnosis, care during and after cancer treatment, comorbidity management, and palliative care. Primary prevention through lifestyle behavioral changes and early detection in the asymptomatic phase remain the most effective strategies to reduce cancer morbidity and mortality. However, structural barriers within the Romanian primary healthcare system, specifically fragmented digital infrastructure, administrative workloads and limited patient health literacy, frequently restrict these preventive activities. The aim of this narrative review is to analyze cancer prevention and management within Romania’s current demographic and socio-economic context, comparing the national system to models from the UK, the Netherlands, and Spain. The analysis highlights operational gaps, including low breast, cervical, and colorectal screening uptake in Romania. We concluded that optimizing patient care requires transitioning to an integrated pathway, supported by practical reforms like digital fast-track scheduling modules managed directly by family physicians.

## 1. Introduction

Family Medicine plays a crucial role within the primary healthcare system, serving as the initial point of contact for individuals seeking medical assistance. As the first representatives of medical structures in contact with patients, family physicians have a significant role in addressing various aspects of health, including the multifactorial etiology of neoplastic diseases. They are positioned to recognize and address potential risk factors contributing to neoplastic diseases, including genetic predisposition, viral infections, smoking, alcohol consumption, stress level, nutritional deficiencies (like vitamin D3 deficiency), and exposure to harmful substances, such as toxic chemicals, pesticides, and heavy metals. Additionally, they conduct screenings and initial diagnosis suspicion, offer medical care throughout and after cancer treatments, assess and manage comorbidities, and provide palliative care. These functions underscore the pivotal role of primary healthcare in addressing the challenges posed by the escalating incidence of neoplastic diseases, contributing to prevention, early intervention, and comprehensive patient care [1,2].

The incidence of neoplastic diseases has consistently risen over the past few decades, attributed to factors such as global population aging, environmental influences, and advancements in screening procedures. According to the International Agency for Research on Cancer (IARC) and the World Health Organization (WHO), global cancer incidence and mortality continue to pose an immense public health challenge [3]. In Romania, recent data from the National Institute of Public Health (INSP) published in 2024 and 2025 indicate a sharp 21% increase in newly registered cancer cases, pushing the total number of oncologic patients under active medical surveillance to an unprecedented 564,764 individuals. This highlights an urgent need for optimized early diagnosis and health care, including primary care pathways [4].

This review provides a comprehensive analysis of the current evidence regarding cancer epidemiology, preventive strategies, clinical pathways and systemic challenges surrounding oncologic disease management in Romania, highlighting family medicine as a key component of multidisciplinary cancer care. The aim of this narrative review is to analyze the current evidence regarding the role of family physicians throughout the oncologic care continuum in Romania, including cancer prevention, screening, early diagnosis, treatment support and palliative care. Particular emphasis is placed on identifying the current barriers limiting the integration of primary care into multidisciplinary oncology services and discussing potential strategies to strengthen the contribution of family medicine within the Romanian healthcare system. 

## 2. Material and Methods 

This study was conducted as a narrative review. A structured search was conducted in the PubMed/MEDLINE database to identify relevant peer-reviewed articles published between January 2016 and May 2026. The search strategy combined Medical Subject Headings (MeSH) and free-text terms adapted to the Romanian healthcare context, including “family medicine”, “primary care”, “general practitioner”, “oncological care”, “cancer screening”, “cancer prevention”, and “Romania”, using the Boolean operators AND and OR. Searches were limited to publications in English and Romanian. 

Additional epidemiological, demographic, screening, and healthcare system data were retrieved from official reports published by the Romanian National Institute of Public Health (INSP), the National Institute of Statistics (INS), Eurostat, the Organisation for Economic Co-operation and Development (OECD), the World Health Organization (WHO), the International Agency for Research on Cancer (IARC) and the CONCORD Programme database managed by the London School of Hygiene and Tropical Medicine. These OECD country profiles function as secondary data sources, synthesizing health indicators collected from Eurostat, national databases, and regional cancer registries.

Publications were considered eligible if they addressed cancer prevention, screening, early diagnosis, treatment support, palliative care, healthcare organization and the role of family physicians and primary care in oncology. Particular emphasis was placed on studies conducted in Romania and on publications directly applicable to the Romanian healthcare system. International publications were additionally included when they provided evidence regarding the organization of primary care in oncology or served as a comparator for Romanian healthcare practice. Exclusion criteria were conference abstracts without full-text publication, non-peer-reviewed commentaries, general oncology studies without a primary care perspective and the grey literature lacking verifiable epidemiological or healthcare data.

Because the present review specifically aimed to analyze the Romanian healthcare context, only studies directly addressing cancer prevention, screening, diagnosis, management or healthcare organization within Romanian primary care are synthesized in Table 1.

Studies were selected according to their relevance to the objectives of the review, methodological rigor, clinical applicability, and contribution to understanding the role of family medicine in oncology. Publications authored by members of the research team were retained only when they fulfilled the same predefined eligibility criteria as all other included studies and contributed unique regional data relevant to the review objectives. 

Given the narrative nature of this review and the heterogeneity of the included evidence, a formal risk-of-bias assessment was not performed. Instead, emphasis was placed on the methodological quality, relevance and applicability of the selected publications to the Romanian primary care setting. 

## 3. Demographic and Socio-Economic Context of Oncologic Disorders in Romania 

The demographic and socioeconomic baseline provides a critical framework for understanding the challenges faced by primary care physicians in cancer management. The updated socio-economic profile for Romania, compiled from the latest Eurostat and National Institute of Statistics (INS) databases, is presented in Table 2.

**Figure 1:**
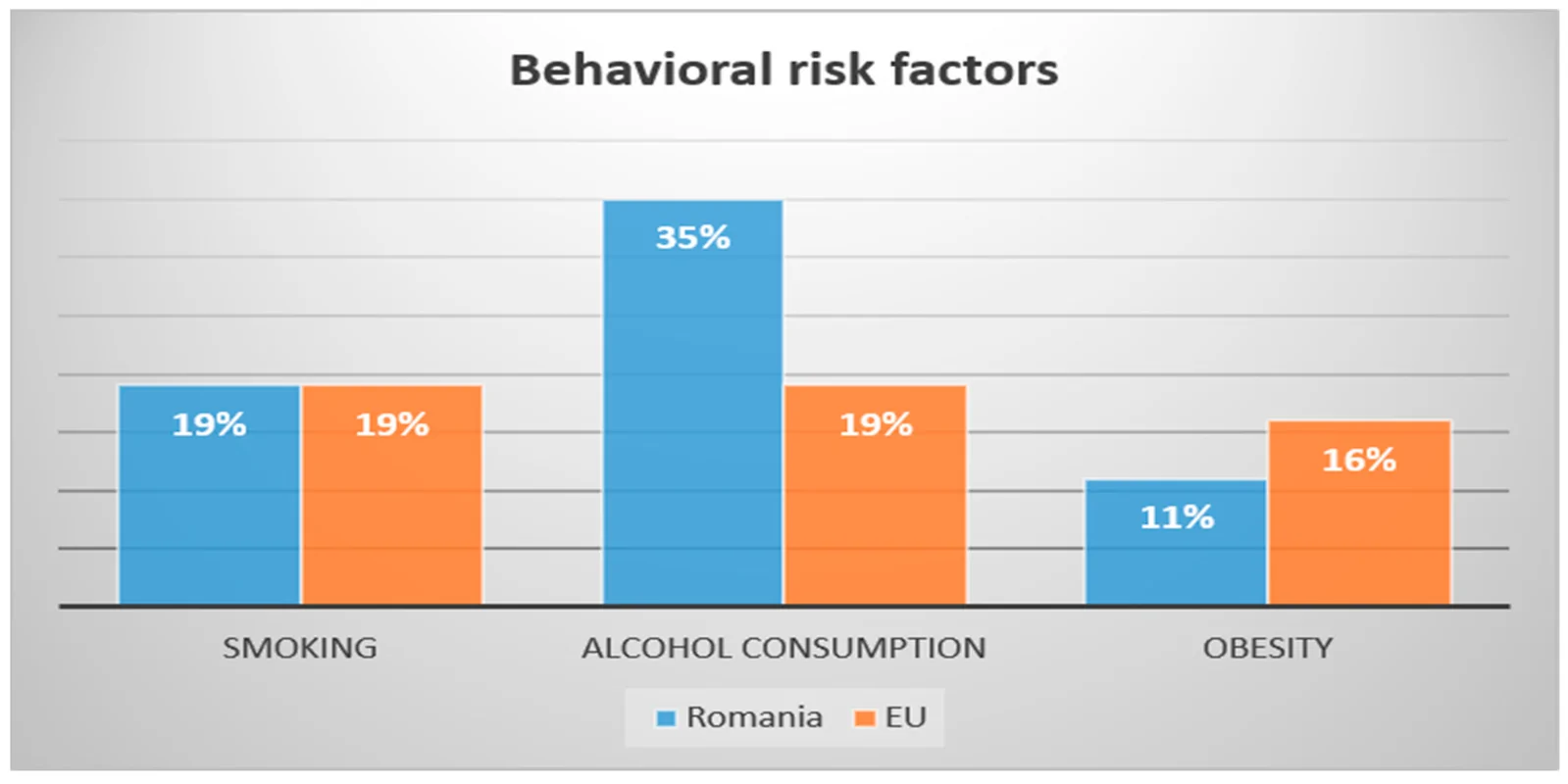
Prevalence of behavioral risk factors for oncologic diseases in Romania compared to Europe (adapted from OECD. Romania: Country Health Profile 2023 [14]). Note: Data represent age- and sex-standardized estimates derived from the European Health Interview Survey (EHIS). Definitions applied: Smoking includes daily tobacco smokers aged 15 and over; alcohol consumption reflects heavy episodic drinking (consuming 60g or more of pure alcohol on a single occasion at least once a month); BMI categories are based on self-reported height and weight, where overweight/obesity is defined as a Body Mass Index (BMI) ≥ 25 kg/m^2^.

Average life expectancy at birth in Romania is around 75.3 years, while the highest life expectancy is 83.2 reported in Spain. Although the lifespan increased over the last two decades, it still ranks among the lowest in the European Union, remaining nearly 5 years below the EU average and only one year more than the last position in the European ranking (74.3 years). A notable gender disparity persists, with women living nearly 8 years longer than men (79.3 years for women compared to 71.5 years for men). This discrepancy is largely driven by significantly higher exposure to behavioral risk factors among men, including higher smoking rates and heavy episodic alcohol consumption [14]. Taking into account the risk factors, although the daily smoking rates among Romanian adults align with the EU average, Romania exhibits nearly double the average alcohol consumption compared to the EU. However, on a positive note, Romania has one of the lowest obesity levels in the EU (Figure 1) [14,15]. Despite lower obesity levels—which theoretically reduce the individual risk for specific obesity-related malignancies—overall cancer mortality remains high. This discrepancy is driven by other prominent behavioral risks, such as heavy episodic alcohol consumption (35% vs. 19% EU average) and high smoking rates, compounded by systemic barriers like delayed diagnosis and restricted access to innovative treatments. 

The primary cause of mortality in Romania is represented by cardiovascular diseases, mostly heart attacks and strokes, accounting for more than half of all deaths (52%), slightly higher than that in other European countries, in which they account for an average of 45% of all deaths [16,17]. The shift towards cancer as the most prevalent cause of death in numerous high-income countries is a consequence of significant reductions in cardiovascular disease mortality, likely attributed to improved prevention and treatment [18,19].

**Figure 2:**
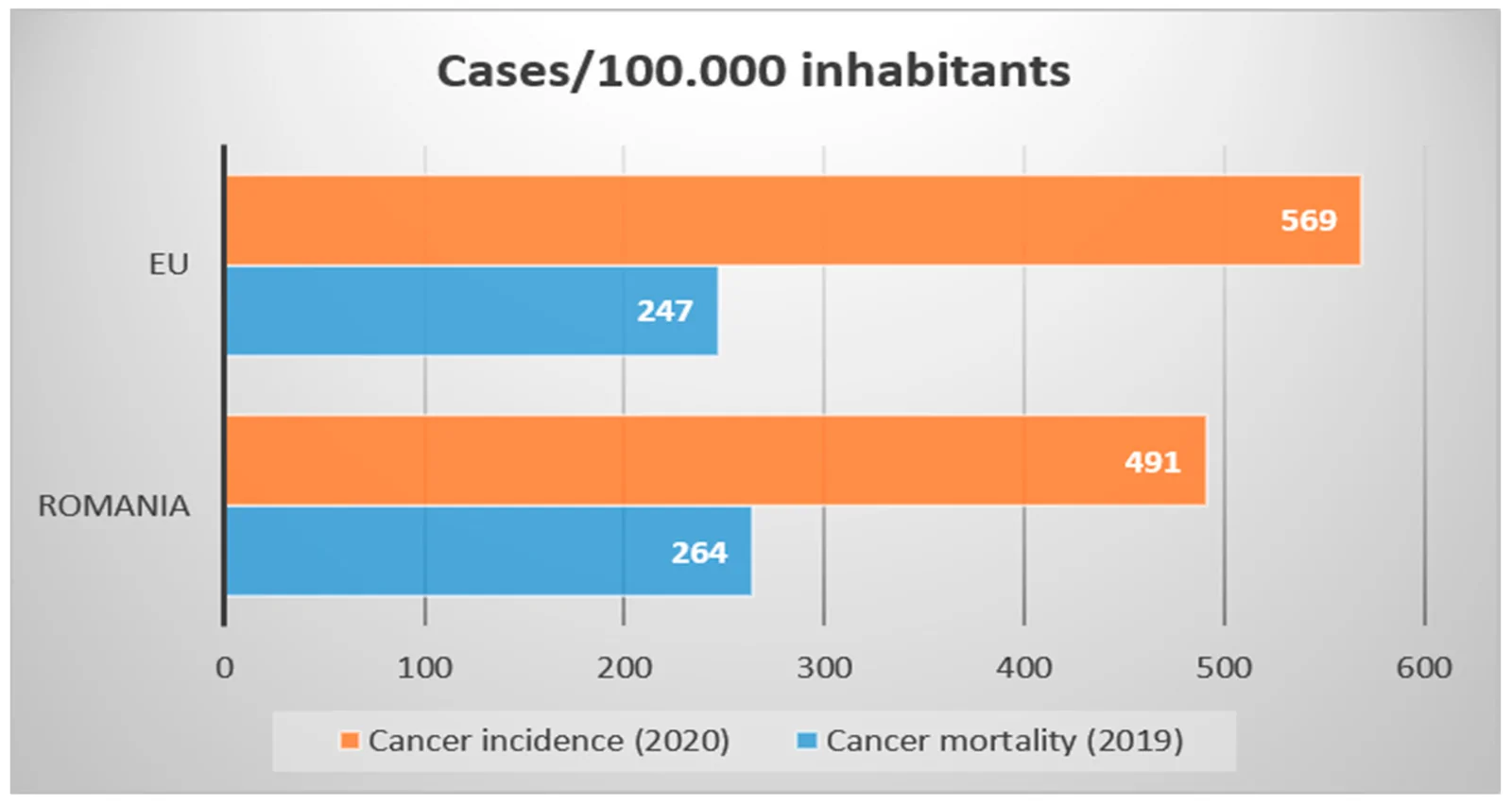
Cancer incidence and mortality in Romania compared to the EU (adapted from [20]). Note: Incidence data from 2020 are compared with 2019 mortality baseline data in alignment with the European Cancer Information System (ECIS) reporting framework. This specific multi-year baseline was utilized by international registries to prevent methodological distortions and underreporting artifacts in cancer-specific mortality tracking caused by the COVID-19 pandemic in 2020.

In 2020, Romania reported a lower cancer incidence rate than the European average, yet its mortality rate was higher, at an estimated 264 vs. 247 deaths per 100,000 individuals (Figure 2) [20]. However, it must be emphasized that these regional comparisons are mostly based on crude incidence measurements. Given that Romania has a younger population demographic than the EU average—with a lower share of the population aged over 65 (19.5% vs. 21.1%, as detailed in Table 2)—using crude rates rather than age-standardized incidence rates (ASIR) represents a conceptual limitation, as age strongly influences absolute oncological risk. While this disparity is associated with underperforming screening systems, Romania’s elevated cancer mortality is multifactorial. Beyond screening, it is driven by advanced stage at diagnosis, delayed access to novel treatments, and geographic variations in healthcare quality. Additionally, these comparative metrics are heavily confounded by structural discrepancies between Romania and the EU, specifically regarding incomplete cancer registration completeness, differences in population-based screening coverage and varying local diagnostic practices.

## 4. Neoplastic Diseases Burden in Romania

In 2022, Romania reported 104,661 new cases of neoplastic diseases and 56,216 deaths, representing almost 17% of all deaths. Methodologically, these numbers integrate two independent data sources: IARC/GLOBOCAN provides modeled statistical estimates with a 2-to-3-year reporting lag [3], while the National Institute of Public Health (INSP) collects raw, official national registry data [4]. The leading cause of cancer mortality across these frameworks was lung cancer (18.9% of cancer-related deaths), followed by colorectal (13.3%) and breast cancer (7.1%) [3,14]. 

In terms of gender-specific disease burden, distinct variations dictate clinical priorities for primary care systems:

- *Male Mortality Trajectory*—The highest cancer-related mortality rates among Romanian men are heavily dominated by lung malignancies (accounting for 23.84% of male oncologic deaths), followed sequentially by colorectal cancer (13.53%), prostate cancer (8.79%), liver cancer (6.89%), stomach cancer (6.50%), and pancreatic cancer (5.40%)
- *Female Mortality Trajectory*—For the female population, breast cancer represents the leading cause of oncologic mortality (16.75%), followed closely by colorectal cancer (12.90%), lung cancer (12.04%), cervical cancer (7.74%), pancreatic cancer (6.29%), and liver cancer (5.43%) [3].

Mortality rates for bladder, pancreatic, prostate, breast, liver, and colorectal cancers experienced an upswing between 2011 and 2019, with a slight decrease noted in the lung and gastric cancer death rates during this 2011–2019 timeframe. Despite the downward trend, the declines were less significant than those witnessed in Europe. Specifically, Romania saw a 3% decrease in lung cancer mortality, compared to the 9% reduction across Europe, and a 17% decrease in gastric cancer mortality over the past decade, although still 60% higher than the European average in 2019 [20].

## 5. Management of the Oncological Patient in Primary Care in Romania

### 5.1. Current Daily Practice

In the Romanian healthcare system, the day-to-day clinical reality of family physicians regarding oncologic patients is highly influenced by many bureaucratic procedures such as issuing repetitive medical prescriptions for reimbursed oncological adjuvants, validating medical leave certificates and formatting physical referral sheets required for specialists, and also the high patient-to-doctor ratios [5,14]. 

In daily practice, family physicians’ clinical intervention is predominantly reactive, centering on the management of acute treatment side effects (including post-chemotherapy emesis, bone marrow suppression, and radiation-induced dermatitis) and the control of chronic comorbidities during active cancer therapy [21,22]. Furthermore, due to the absence of formal communication pathways connecting primary care to hospital-based Tumor Boards, Romanian family physicians are left to decode complex tertiary discharge summaries delivered physically by the patients, without direct access to the treating specialist’s clinical decision-making process [2,5].

### 5.2. Operational Barriers in Primary Care 

Data from a national survey assessing Romanian family physicians’ perspective on personalized cancer prevention quantifies the specific operational constraints within primary care, such as the lack of targeted post-graduate professional training in modern oncology interfaces, genomics, and personalized risk stratification. Furthermore, the survey highlighted absence of collaborative networks; family physicians operate in digital fragmentation, completely cut off from multidisciplinary oncology platforms, addressing these documented deficits in professional education, digital integration and consultation scheduling [5]. 

### 5.3. Comparative European Framework

In order to accurately assess the effectiveness of oncology integration in Romanian primary care, it is useful to compare it with well-established European models from countries where the role of the family physician is better legally and practically defined. The main characteristics of primary care pathways of oncologic patients from the United Kingdom, the Netherlands and Spain compared with Romania are showed in Table 3. 

## 6. Cancer Prevention in Primary Care 

Cancer prevention in primary care should focus on the identification and modification of environmental, occupational, behavioral, and metabolic risk factors associated with cancer development. Family physicians are uniquely positioned to evaluate cancer risk, offer preventive counseling, and advocate for evidence-based interventions, including vaccination, routine screening, and healthy lifestyle recommendations. Adopting preventive measures and managing lifestyle risk factors can prevent an estimated 30% to 50% of cancer cases [5].

Modifiable risk factors account for a substantial proportion of the cancer burden. However, inadequate public awareness and limited knowledge regarding these risk factors continue to hinder effective cancer prevention [6]. Routine primary care consultations provide opportunities for family physicians to counsel patients on modifiable cancer risk factors, including smoking, alcohol consumption, unhealthy diet, physical inactivity, obesity, and occupational exposures. They also assess hereditary cancer risk and promote participation in evidence-based screening programs [13]. 

## 7. Oncologic Screening Programs Implemented in Romania

**Figure 3:**
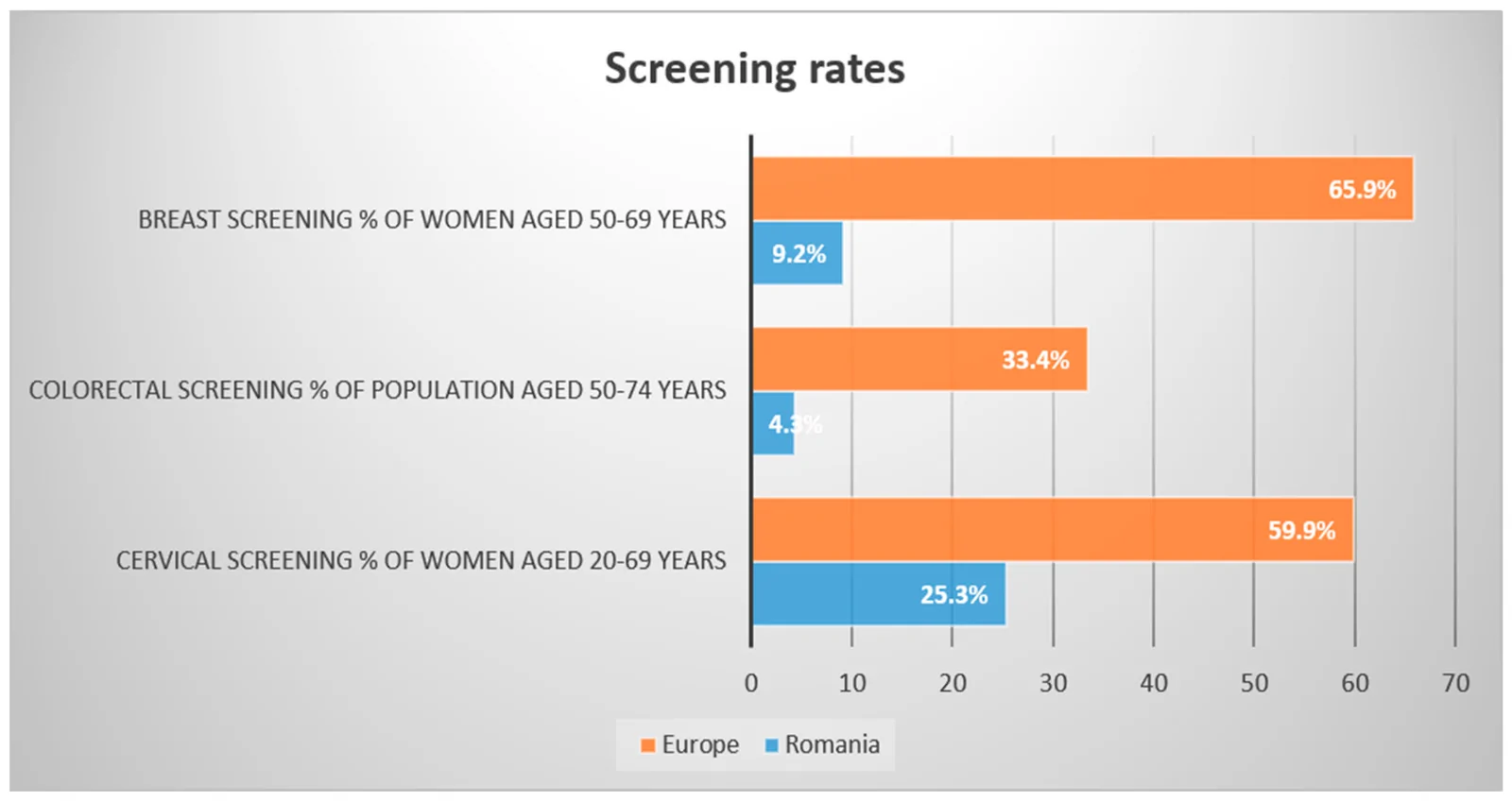
Screening rates for breast, colorectal, and cervical cancers in Romania and Europe (adapted from OECD. EU Country Cancer Profile: Romania 2023 [20]).

Starting with 2018–2019, significant investments were directed toward developing population-based screening programs for cervical, breast, and colorectal cancers. However, challenges in implementing systematic screening programs have led to low participation rates compared to the European average. Despite formal guidelines involving family physicians in coordinating preventive screening schedules, overall population adherence remains suboptimal (Figure 3).

The national screening program for cervical cancer was introduced in 2012, with women aged 25 to 64 years being eligible for a smear test every five years, regardless of their health insurance status. The data indicated that only 12% of eligible women utilized the screening program until 2017, while an additional 30% underwent opportunistic testing [10]. Cervical cancer screening in Romania has the lowest rate in Europe according to the Country Cancer Profile of 2023, showing that only 25.3% of women aged 20–69 years have undergone a cervical smear test in the past three years, which is less than half the European average of 59.9%. Screening participation reveals significant demographic gaps. Attendance is 2.3 times higher for women in urban areas, 3.3 times higher for those with higher incomes, and 4.6 times higher for women with higher education levels [20]. To overcome these discrepancies and modernize screening strategies, regional programs such as the Screening for Cervical Cancer and Early Treatment (SCCET) project have adopted primary high-risk HPV DNA testing. The program results from the South Region suggest that integrating molecular testing with public education significantly improves population participation [9].

The national screening programs for breast and colorectal cancers are in the early stages of implementation. In 2018, Romania started a pilot program for breast cancer screening, but in 2019, only 9.2% of women aged 50 to 69 reported having a mammogram within the past two years. This represents the lowest rate among European countries, which have an average of 65.9%. A similar socio-economic discrepancy is observed for breast cancer screening, where women with higher income and those residing in urban areas show a significantly higher uptake [20]. 

Romania has recently initiated colorectal cancer screening for the population aged 50 to 74, making it one of the last countries in Europe to do so. In 2019, only 4.3% of the population aged 50 to 74 in Romania had been tested for colorectal cancer within the past two years, compared to the European standard of 33.4% [20]. The discrepancy between income groups is negligible here. The ROCCAS (Romania Colorectal Cancer Awareness & Screening) project was launched in 2019, while its four regional pilot colorectal cancer screening programs were implemented in 2020 [11]. Within the ROCCAS program, family physicians used standardized questionnaires for colorectal cancer risk stratification. Average-risk individuals underwent fecal immunochemical test (FIT) screening, while symptomatic or high-risk patients were referred directly for colonoscopy and specialist assessment. This approach highlights the central role of primary care in early cancer detection [7,12].

Through Law No. 293/2022, Romania formally launched the National Cancer Control Plan (PNCC 2023–2030) to shift from fragmented, pilot-scale screening to long-term, population-based programs [8,26]. The PNCC fundamentally redefines national cancer policies by prioritizing the establishment of organized screening registries and multi-annual funding pathways. However, translating this national strategy into primary care practice exposes family physicians to significant digital and educational challenges. Specifically, these practitioners encounter a major lack of interoperable software, postgraduate training deficits and an absence of organized collaborative oncology networks when attempting to operationalize early detection frameworks [5]. Furthermore, policy recommendations must actively account for regional variations in health and cancer literacy across the population to ensure optimal participation in organized screening initiatives [6].

## 8. Treatment and Long-Term Monitoring of Oncologic Patients

In Romania, the healthcare system relies on social health insurance. Cancer care is guaranteed and cost-free, regardless of their insurance status. The National Oncology Program delivers a wide range of cancer treatments through inpatient care or one day hospitalization. These therapies include chemotherapy, immunotherapy, hormone therapy, targeted radiation, surgery, and bone marrow or stem cell transplants.

Despite healthcare and treatment being free for the Romanian population regarding cancer care, barriers persist due to difficult access and an uneven distribution of medical services, resulting in patients paying out-of-pocket for private services to receive more prompt access. Most out-of-pocket spending goes toward medications, though patients also face direct costs for various diagnostic procedures.

Romania lags behind the rest of Europe in providing access to new cancer treatments. While the National Health Insurance House updates its list of covered medications periodically, there is a substantial delay between a drug receiving European Medicines Agency (EMA) approval and becoming locally reimbursed. Between 2018 and 2021, EMA approved 168 new molecules, but only 51 were included in the national list by January 2023. The time-period between obtaining marketing authorization and patient availability extended up to 903 days, making it one of the longest lag periods in Europe [27].

Furthermore, the structural architecture of the PNCC 2023–2030 introduces modern concepts such as specialized patient navigators and multidisciplinary Tumor Boards to optimize therapeutic pathways. Within this shifting paradigm, the continuous integration of primary care remains a subject of ongoing academic and clinical discussion. Ensuring a well-defined clinical interface between family medicine and tertiary oncology platforms is essential to prevent coordination deficits once patients transition from active hospital-based treatment back to long-term community-based surveillance [1,2,21].

### 8.1. Long-Term Monitoring of Cancer Patients in Primary Care

Cancer survivors require continuous follow-up after completion of active treatment to identify disease recurrence, second primary malignancies, and persistent or delayed treatment-related complications. Follow-up care must encompass assessments of physical symptoms, functional status, quality of life, and treatment-related toxicities, all of which can evolve and vary based on the type of cancer and therapeutic modality [21,22,28].

Medication review represents an integral component of long-term follow-up, particularly in patients receiving multiple concomitant therapies, as polypharmacy increases the risk of clinically relevant drug–drug interactions and adverse events [29,30]. Primary care follow-up should integrate the assessment of psychological distress, anxiety, and depressive symptoms, together with surveillance for late complications, including cardiovascular toxicity, which may occur months or years after cancer treatment. Identification of these conditions allows for timely referral to oncology, cardiology, psychology, or psychiatry services whenever additional evaluation or treatment is required [22,31].

### 8.2. Palliative and End-of-Life Care for Oncologic Patients

Within the Romanian healthcare system, family physicians can support patients with cancer from the time of diagnosis by providing basic palliative care, including symptom relief and pain management and by coordinating referral to specialized palliative care services when needed.

In Romania, palliative care inpatient beds are available in various settings, including public and private hospitals, totaling 1779 beds nationwide. Of these, 59% are provided free of charge, while the remainder require payment, making them inaccessible to a part of population. Recent efforts have been made to enhance palliative care capacity, with the Health Program 2021–2027 earmarking funds to improve palliative care infrastructure, particularly in regions with significant deficits [20].

## 9. Quality of Care and Survival Rate of Oncologic Patients in Romania

**Figure 4:**
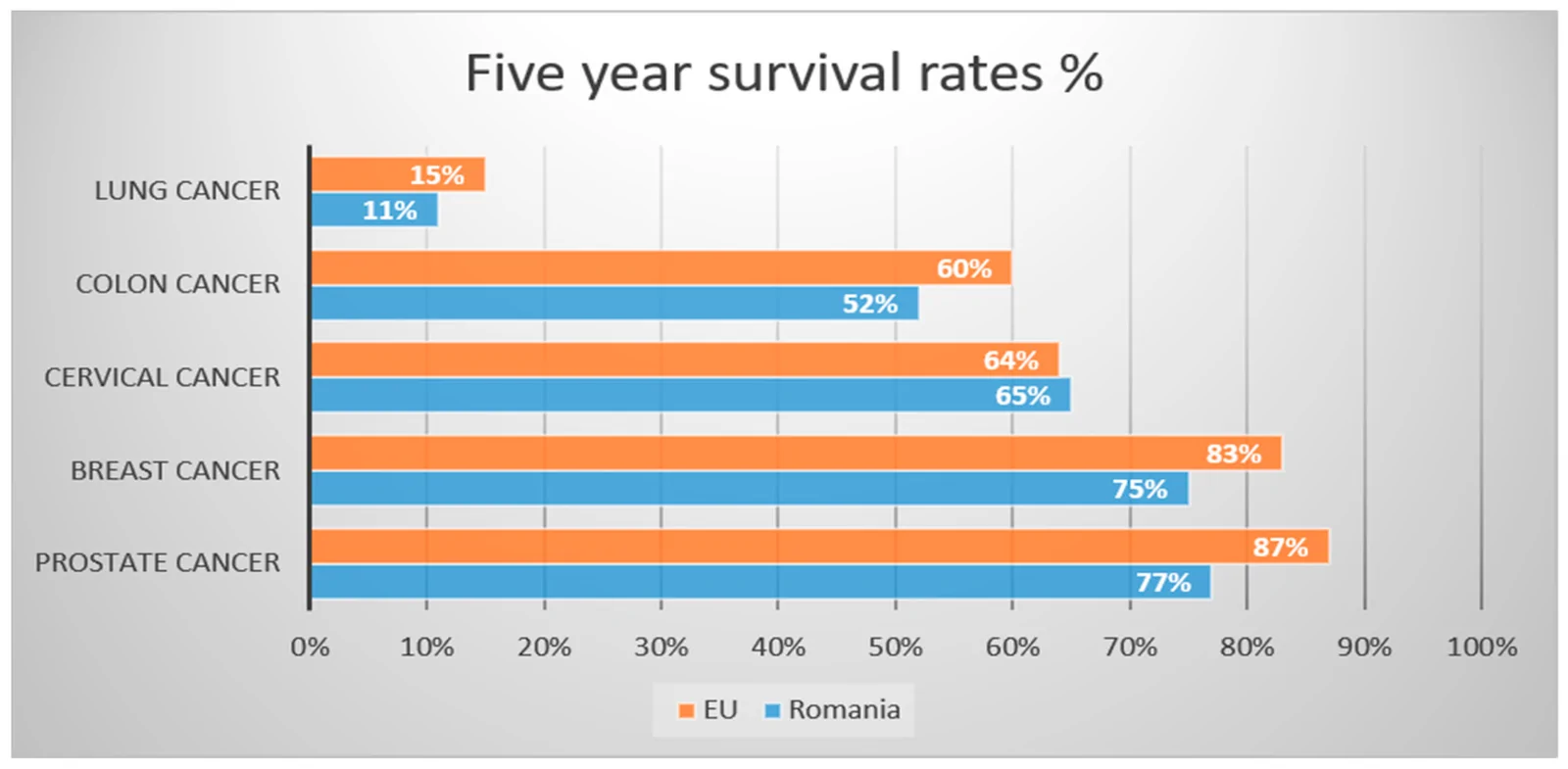
Five-year survival rates for top frequent cancers—data refer to people diagnosed between 2010 and 2014. (Adapted from OECD. EU Country Cancer Profile: Romania 2023 [20].)

A significant indicator of the quality of care is the 5-year survival rate, which is notably lower for the most common cancers in Romania compared to Europe. Prostate cancer exhibits a 77% survival rate, in contrast to Europe’s 87%, while breast cancer shows a 75% survival rate compared to Europe’s 83%. Despite an increase from 8% to 11% in 2014, the 5-year survival rate for lung cancer still significantly trails behind the European average of 15%. Childhood leukemia has a 5-year survival rate of only 54%, which is 28% lower than in any other European country, according to the CONCORD Programme registry surveillance [32]. The survival rate for cervical cancer in Romania is 1 percentage point above the European average, but the prevalence is much higher, and screening rates are much lower, potentially leading to underdiagnosis. The COVID-19 pandemic disrupted numerous screening programs and delayed patient presentation for diagnostic and therapeutic procedures, potentially compromising early detection and long-term survival rates [33]. These outcomes underscore the critical need for timely screening, early diagnosis, and therapeutic interventions, with a distinct focus on enhancing overall clinical effectiveness (Figure 4) [20].

**Figure 5:**
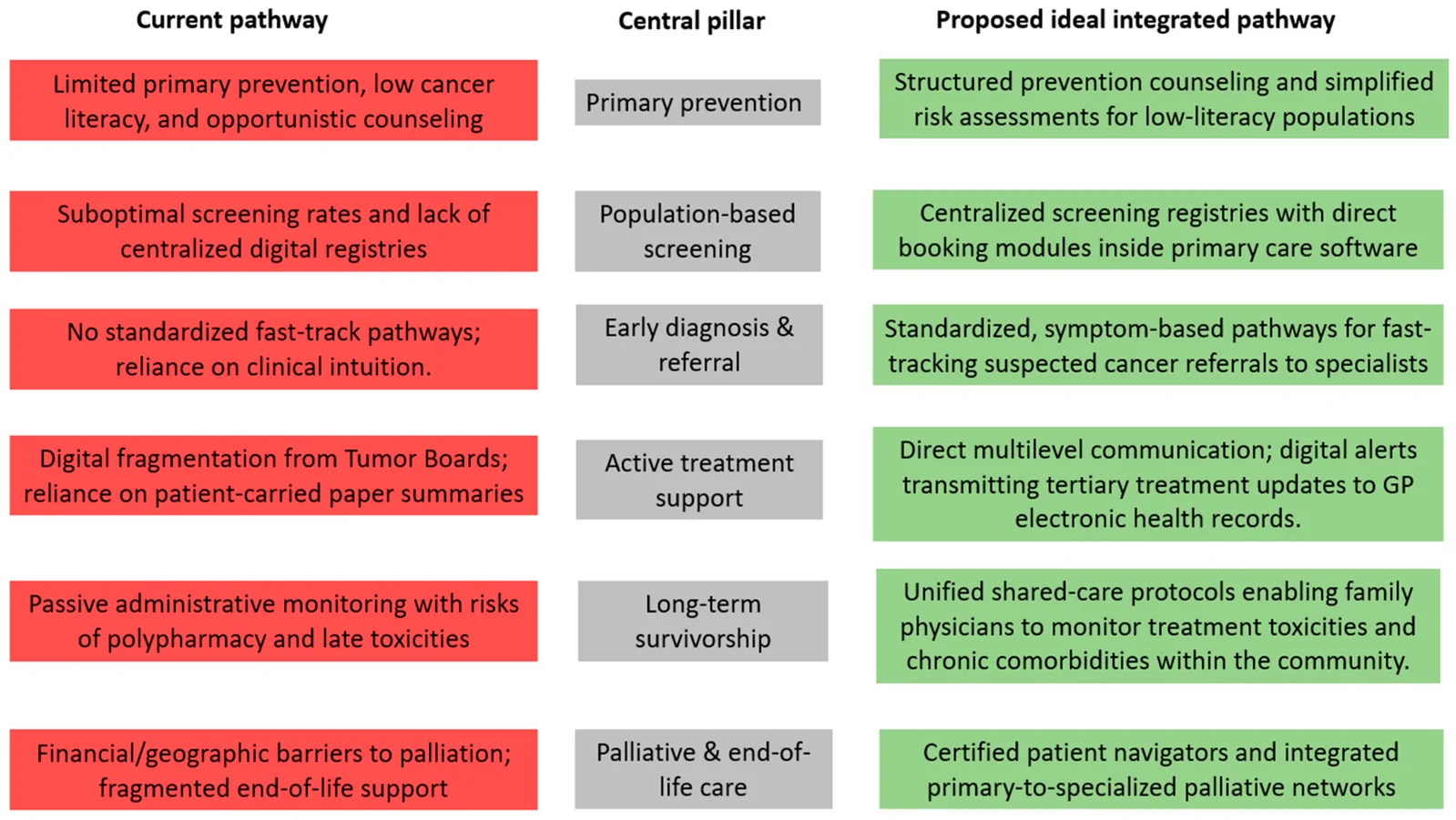
Proposed framework illustrating the role of family physicians across the cancer care continuum in Romania: current practice and proposed integrated model. Red boxes represent limitation of the current pathway, grey boxes represent the central pillars of cancer care and green boxes represent the proposed integrated pathway.

To address the systemic fragmentation identified across the Romanian oncological landscape, we propose a transition from the current episodic care model to an optimized, evidence-based integrated workflow. Figure 5 outlines this conceptual framework, mapping the entire oncology continuum across six core clinical stages: from primary prevention to end-of-life care. By contrasting the current structural bottlenecks (red boxes)—such as fragmented digital infrastructure, low health literacy, and opportunistic screening—with the proposed ideal pathways (green boxes), this model demonstrates how the strategic integration of family physicians can bridge the gap between community medicine and tertiary oncology networks.

Our review has several limitations, mainly: few available publications and limited data referring to the role of family physicians in management of oncologic diseases in Romania and differences in cancer registration, screening coverage and diagnostic practices between Romania and other EU countries. 

## 10. Conclusions

We concluded that, while the National Cancer Control Plan led to significant legislative and infrastructural changes in Romania, optimizing clinical outcomes requires a shift from general mandates to resource-based primary care pathways. According to comparison with well-established healthcare systems from other European countries, family physicians’ structural and professional empowerment continues to be essential in reducing the national cancer burden and improving long-term patient survival. 

To bridge the gap between policy design and clinical reality, future healthcare strategies should prioritize targeted, data-driven interventions over broad policy goals. Some recommendations for improvement could be retrieved from this review. 

Since family physicians are designated as screening coordinators for National Screening Programme of breast cancer, it is recommended to integrate interoperable, fast-track diagnostic scheduling modules directly into primary care software. 

Similarly, reducing regional healthcare disparities requires combining community-based health literacy initiatives with localized patient navigation frameworks to prevent high-risk, vulnerable populations from dropping out of the care continuum. Finally, to bridge the communication gap during post-treatment monitoring, establishing standardized shared-care protocols and digital feedback circuits between primary care practices and hospital-based Tumor Boards is essential. Ultimately, providing focused training to primary care physicians, strengthening digital integration and establishing clear communication channels is expected to facilitate earlier diagnosis, improve continuity of care and support high-quality oncology care across all patient populations in Romania. 

## Figures and Tables

**Table 1:** Relevant studies evaluating prevention, diagnosis and management of oncologic diseases in Romania.

First Author, Year	Study Design	Main Focus	Implications for Primary Care	Limitations
Nicoară D, 2025 [5]	Cross-sectional survey	Personalized cancer prevention	Identifies specific gaps in post-graduate training (genomics, risk stratification) and highlights the critical need to overcome digital fragmentation in primary care workflows.	Relies on self-reported survey data; lacks longitudinal tracking of actual clinical outcomes.
Coman MA, 2026 [6]	Policy analysis	Health and cancer literacy	Positions the family physician as a primary health educator to address structural deficits in cancer literacy and prevent patient drop-out from screening initiatives.	Focuses on broad policy frameworks without primary quantitative interventional data
Manuc M, 2024 [7]	Multicentre pilot study	Colorectal cancer screening (ROCCAS)	Validates the clinical utility of GP-led risk stratification using standardized questionnaires combined with direct fecal immunochemical testing (FIT) coordination.	Limited by pilot-phase implementation scope and regional logistical variations.
Bohiltea RE, 2022 [8]	Review	Oncologic screening	Contextualizes the evolving operational mandates for general practitioners under the strategic legal framework of the new National Cancer Control Plan (PNCC)	Narrative review methodology without original patient cohort data.
Saveliev GM, 2025 [9]	Observational study	HPV screening	Demonstrates that integrating primary high-risk HPV DNA testing with targeted public education successfully increases screening participation tracking.	Regionally specific cohort data (South Region), limiting immediate nationwide generalization
Todor RD, 2021 [10]	Multicentric cross-sectional study	Cervical cancer	Emphasizes the historical underutilization of organized screening frameworks, underscoring the limitations of relying purely on opportunistic testing.	Cross-sectional design restricting causal inferences regarding screening dropouts.
Gheorghe C, 2023 [11]	Policy analysis	Colorectal cancer screening	Provides evidence-based programmatic lessons from the ROCCAS pilot to guide the transition from fragmented to organized, large-scale screening pathways	Policy-level evaluation lacking detailed individual patient-level clinical outcomes.
Bocioaga AG, 2026 [12]	Retrospective study	Colorectal cancer screening in S-W Region	High detection rate of precancerous lesion empowers family physicians to persuade hesitant patients to undergo follow-up colonoscopies	Retrospective single-region design subject to local referral biases
Iancu MA, 2021 [13]	Review	Early diagnosis of colorectal cancer	Lifestyle counselling, assessment of hereditary cancer risk, and early referral of symptomatic patients from primary care.	Narrative scope lacking primary quantitative data collection.

**Table 2:** Demographic and socio-economic context—Romania, 2022 (OECD. Romania: Country Health Profile 2023 [14]).

	Romania	European Union
**Demographic Factors**		
Population size	19,042,455	446,735,291
Share of population over age 65 (%)	19.5%	21.1%
Fertility rate ^1^ (2021)	1.8	1.5
**Socio-economic Factors**		
GDP per capita ^2^ (EUR PPP)	27,073	35,219
Relative poverty rate ^3^ (%)	21.2	16.5
Unemployment rate (%)	5.6	6.2

^1^ Number of children born per woman aged 15–49. ^2^ Purchasing power parity (PPP) = the rate of currency conversion that equalizes the purchasing power of different currencies by eliminating the differences in price levels between countries. ^3^ Percentage of persons living with less than 60% of median equivalized disposable income. Bold text indicates category headings within the table.

**Table 3:** Structural comparison of primary care oncology integration across distinct European healthcare frameworks [2,5,14,23,24,25].

Country	Early Detection & Referral Pathways	Digital Integration & Interoperability	Post-Treatment Surveillance & Shared-Care	Cross-Tier Communication (Tumor Boards)
**United Kingdom [23]**	Standardized, symptom-based suspected cancer referral pathways guided by NICE NG12 and monitored through the NHS Faster Diagnosis Standard	Partially integrated; localized networks with standardized electronic referral templates.	Structured transition protocols; active GP role in early survivorship and monitoring.	Indirect but standardized reporting via Multi-Disciplinary Team (MDT) electronic outcomes.
**Netherlands [24]**	Protocol-driven risk stratification co-developed by the Dutch College of General Practitioners (NHG).	Regional GP-led survivorship models supported by individual care plans and regular oncology meetings with hospital specialists.	Formalized shared-care guidelines for community-based long-term survivorship management.	Regular oncology meetings support shared responsibility and a clearer transfer from hospital to primary care.
**Spain [25]**	The national strategy recommends coordinated and protocolized follow-up between primary and hospital care, with implementation varying across autonomous communities	Interoperable regional platforms allowing shared access to hospital-tier clinical data.	The national strategy recommends coordinated and protocolized follow-up between primary and hospital care, with implementation varying across autonomous communities	Regional pathways and coordination guides support communication between primary care and hospital specialists.
**Romania [2,5,14]**	Absent standardized fast-track pathways; referrals depend on subjective clinician intuition.	High fragmentation; lack of interoperable cross-tier digital platforms	Passive administrative surveillance; lack of formalized, evidence-based shared-care protocols.	No formalized communication channels; relies heavily on patient-carried paper summaries.

## Data Availability

No new data were created or analyzed in this study. Data sharing is not applicable to this article.

## References

[1] Cheung W.Y., Aziz N., Noone A.M., Rowland J.H., Potosky A.L., Ayanian J.Z. (2013). Physician Preferences and Attitudes Regarding Different Models of Cancer Survivorship Care: A Comparison of Primary Care Providers and Oncologists. J. Cancer Surviv..

[2] Lawrence R.A., McLoone J.K., Wakefield C.E., Cohn R.J. (2016). Primary Care Physicians’ Perspectives of Their Role in Cancer Care: A Systematic Review. J. Gen. Intern. Med..

[3] Ferlay J., Ervik M., Lam F., Laversanne M., Colombet M., Mery L., Soerjomataram I., Bray F. (2024). Global Cancer Observatory: Cancer Today.

[4] Information Bulletin—Main Indicators of Health Status in Romania. https://insp.gov.ro/centrul-national-de-statistica-in-sanatate-publica-cnssp/date-statistice-pagina-de-descarcare/.

[5] Nicoara D., Cristescu C., Pop I.C., Ilies R.A., Nicoara N., von Stauffenberg A.O., Matei S., Muntean V.M., Achimas-Cadariu P. (2025). Family Physicians’ Perspectives on Personalized Cancer Prevention: Barriers, Training Needs, Quality Improvements and Opportunities for Collaborative Networks. J. Clin. Med..

[6] Coman M.A., Cucu I.A., Ungureanu M.I., Chereches R.M., Florescu S. (2026). Health and Cancer Literacy as a Referral Point in Policy Recommendations for Romania. Discov. Public Health.

[7] Manuc M., Diculescu M., Dumitru E., Gheonea D.I., Jinga M., Ionita-Radu F., Mergeani D., Udrescu M., Manuc T.E., Cotruta B. (2024). Introducing Colorectal Cancer Screening in Romania—Preliminary Results From the Regional Pilot Programs (ROCCAS). J. Gastrointestin. Liver. Dis..

[8] Bohîlţea R.-E. (2022). The National Plan To Prevent and Combat Cancer in Romania, in the Global Context. Rom. J. Prev. Med..

[9] Saveliev G.M., Ciuvică A.I., Cretoiu D., Varlas V.N., Balalau C., Balescu I., Bacalbasa N., Bohiltea L.C., Suciu N. (2025). Screening for Cervical Cancer and Early Treatment (SCCET) Project—The Programmatic Data of Romanian Experience in Primary Screening for High-Risk HPV DNA. Diagnostics.

[10] Todor R.D., Bratucu G., Moga M.A., Candrea A.N., Marceanu L.G., Anastasiu C.V. (2021). Challenges in the Prevention of Cervical Cancer in Romania. Int. J. Environ. Res. Public Health.

[11] Gheorghe C. (2023). The National Screening Program for Colorectal Cancer. Rom. J. Prev. Med..

[12] Bocioaga A.-G., Cretu O.-I., Stepan A.E., Ciurea R.N., Obleaga C., Sacerdoțianu V.-M., Florescu D.N., Gheonea D.I., Florescu M.-M. (2026). Diagnostic Yield and Histopathological Features of Colorectal Lesions Detected Through a Regional Screening Program From the South-West Oltenia Region, Romania. Cancers.

[13] Iancu M.A., Ganea G., Călin R.D., Eremia I.A., Ticărău A., Diaconu C.C., Matei D. (2021). The Role of the Family Doctor in the Early Diagnosis of Colorectal Cancer. Rom. J. Med. Pract..

[14] Romania: Country Health Profile 2023. https://www.oecd.org/publications/romania-country-health-profile-2023-f478769b-en.htm.

[15] Preventing Harmful Alcohol Use. https://www.oecd-ilibrary.org/social-issues-migration-health/preventing-harmful-alcohol-use_6e4b4ffb-en.

[16] Beating Cardiovascular Disease: The Role of Europe’s Environment. https://www.eea.europa.eu/publications/beating-cardiovascular-disease.

[17] Timmis A., Vardas P., Townsend N., Torbica A., Katus H., De Smedt D. (2022). ESC Scientific Document Group. European Society of Cardiology: Cardiovascular Disease Statistics 2021. Eur. Heart J..

[18] Unal B., Erkoyun E., Maximova K., Farrington J., Loyola E., Critchley J., O’Flaherty M., Capewell S. (2017). Impact of NCD Policies on Ischaemic Heart Disease and Premature NCD Mortality Change in Europe: Belgin Unal. Eur. J. Public Health.

[19] Bray F., Laversanne M., Weiderpass E., Soerjomataram I. (2021). The Ever-Increasing Importance of Cancer as a Leading Cause of Premature Death Worldwide. Cancer.

[20] EU Country Cancer Profile: Romania 2023. https://www.oecd-ilibrary.org/social-issues-migration-health/eu-country-cancer-profile-romania-2023_267467c6-en.

[21] Carek S., Emerson J.F., Patel J. (2024). Primary Care of Adult Cancer Survivors. Am. Fam. Physician.

[22] Lee E.M., Jiménez-Fonseca P., Galán-Moral R., Coca-Membribes S., Fernández-Montes A., Sorribes E., García-Torralba E., Puntí-Brun L., Gil-Raga M., Cano-Cano J. (2023). Toxicities and Quality of Life During Cancer Treatment in Advanced Solid Tumors. Curr. Oncol..

[23] National Institute for Health and Care Excellence (NICE) (2015). Suspected Cancer: Recognition and Referral.

[24] van Leeuwen A., Wind J., van Weert H., de Wolff V., van Asselt K. (2018). Experiences of General Practitioners Participating in Oncology Meetings With Specialists To Support GP-Led Survivorship Care: An Interview Study From the Netherlands. Eur. J. Gen. Pract..

[25] Prades J., Espinàs J.A., Font R., Argimon J.M., Borràs J.M. (2011). Implementing a Cancer Fast-Track Programme Between Primary and Specialised Care in Catalonia (Spain): A Mixed Methods Study. Br. J. Cancer.

[26] Law No. 293/2022 on the Prevention and Control of Cancer [Legea nr. 293/2022 pentru prevenirea și combaterea cancerului]. https://legislatie.just.ro/Public/DetaliiDocument/261246.

[27] EFPIA Patients W.A.I.T. Indicator 2022 Survey. https://www.efpia.eu/media/s4qf1eqo/efpia_patient_wait_indicator_final_report.pdf.

[28] Cazacu S.M., Săftoiu A., Iordache S., Ghiluși M.C., Georgescu C.V., Călin C., Burtea E.D., Carțu D., Leru P.M. (2022). Factors Predicting Occurrence and Therapeutic Choice in Malignant Colorectal Polyps: A Study of 13 Years of Colonoscopic PolyPectomy. Rom. J. Morphol. Embryol..

[29] Turossi-Amorim E.D., Camargo B., Schuelter-Trevisol F. (2022). Prevalence of Potential Pharmacological Interactions in Patients Undergoing Systemic Chemotherapy in a Tertiary Hospital. Hosp. Pharm..

[30] Albadour H.M., Salem D.S., Hussain E.A., Osman H., Basiouny L.A., Srrani H., AlAmri O.S., Alshaqi M.A. (2026). Impact of Drug-to-Drug Interactions on Patients With Advanced Cancer in Palliative Care Settings. Cureus.

[31] Fidalgo A.P., Alonso P., Andión M., Cañete A., Collado E., Colino C.G., Codina J.G., Carrasco X.D., Sanz R.G., Expósito S.H. (2026). Management of Late Toxicities and Specific Follow-Up Needs of Adolescent and Young Adult Cancer Survivors: Recommendations From Scientific Societies in Spain. Clin. Transl. Oncol..

[32] Allemani C., Matsuda T., Di Carlo V., Harewood R., Matz M., Nikšić M., Bonaventure A., Valkov M., Johnson C.J., Estève J. (2018). Global Surveillance of Trends in Cancer Survival 2000–2014 (CONCORD-3): Analysis of Individual Records for 37,792,211 Patients Diagnosed With One of 18 Cancers From 322 Population-Based Registries in 71 Countries. Lancet.

[33] Cazacu S.M., Rogoveanu I., Turcu-Stiolica A., Vieru A.M., Gabroveanu A., Popa P., Pirscoveanu M., Cartu D., Streba L. (2025). Impact of the COVID-19 Pandemic on Gut Cancer Admissions and Management: A Comparative Study of Two Pandemic Years to a Similar Pre-Pandemic Period. Healthcare.

